# Functional Genome Analysis for Immune Cells Provides Clues for Stratification of Systemic Lupus Erythematosus

**DOI:** 10.3390/biom13040591

**Published:** 2023-03-25

**Authors:** Keishi Fujio

**Affiliations:** Department of Allergy and Rheumatology, Graduate School of Medicine, The University of Tokyo, Tokyo 113-8655, Japan; fujiok-int@h.u-tokyo.ac.jp

**Keywords:** systemic lupus erythematosus, genome wide association study, functional genome analysis, B cell, OXPHOS, prognosis

## Abstract

Systemic lupus erythematosus (SLE) is caused by a combination of genetic and environmental factors. Recently, analysis of a functional genome database of genetic polymorphisms and transcriptomic data from various immune cell subsets revealed the importance of the oxidative phosphorylation (OXPHOS) pathway in the pathogenesis of SLE. In particular, activation of the OXPHOS pathway is persistent in inactive SLE, and this activation is associated with organ damage. The finding that hydroxychloroquine (HCQ), which improves the prognosis of SLE, targets toll-like receptor (TLR) signaling upstream of OXPHOS suggests the clinical importance of this pathway. IRF5 and SLC15A4, which are regulated by polymorphisms associated with SLE susceptibility, are functionally associated with OXPHOS as well as blood interferon activity and metabolome. Future analyses of OXPHOS-associated disease-susceptibility polymorphisms, gene expression, and protein function may be useful for risk stratification of SLE.

## 1. Introduction

Systemic lupus erythematosus (SLE) is a prototypical autoimmune disease that presents with intractable organ damage as a result of the breakdown of self-tolerance to nuclear antigens. SLE is a highly heterogeneous disease, and the combination of organ involvement varies from patient to patient [1]. In addition, treatment response is also heterogeneous, with 70% undergoing repeated remissions and relapses, 15% experiencing long-term remission, and 15% remaining active [2]. Currently, SLE is treated with glucocorticoids and immunosuppressive agents, however, the long-term prognosis for SLE remains poor, with the median age of death in many SLE cohorts being in the 50s and 60s [3]. A number of epidemiologic studies have demonstrated that organ damage in these patients is associated with atherosclerosis and infection as side effects of glucocorticoids [4]. Therefore, recent treat-to-target strategies for SLE aim to minimize the use of glucocorticoids [5]. However, management of these patients involves the difficult problem that dose reduction of glucocorticoids may induce relapse. Relapse occurs in about a quarter of SLE cases when low-dose glucocorticoids are discontinued [6]. Therefore, to promote successful treatment, it is essential to identify precise immunological pathways associated with remission and high disease activity in order to select suitable therapeutic agents. 

In SLE, following activation of the innate immune system, including macrophages, dendritic cells, and neutrophils, an autoimmune response is formed by the adaptive immune system, including helper T cells, memory B cells, and plasma cells [7,8]. Mitochondrial DNA in neutrophil extracellular traps (NETs) activates cGAS-STING in macrophages and toll-like receptor (TLR)9 in plasmacytoid dendritic cells (pDC) to produce type I interferon (IFN) [9]. Type I IFN is thought to enhance the humoral immune response and induce autoantibody production by activating peripheral helper T (Tph) cells that help B cells outside the follicle [10] and by directory promoting B-cell differentiation into plasma cells [11]. Furthermore, metabolic modification of immune cells via stimulation of innate immune signals and cytokines has also been reported to be involved in the autoimmune response of SLE [12,13].

Although conventional basic immunological analyses have revealed functional relationships among immune cells, it is difficult to determine which immune cells play causal roles. In this context, information on genetic polymorphisms obtained by genome-wide association studies (GWAS) and gene-expression information specific to immune cells provide important clues to understanding which immune cells are important in SLE. Expression quantitative trait loci (eQTL) are genetic variants that affect the expression of one or more genes. The term “eGenes” has been used to describe those genes whose expression levels have been associated with genetic polymorphisms [14]. If the pathophysiology of SLE is enhanced in the presence of high expression of such eGenes, eGene expression and susceptibility-polymorphism genotypes may be biomarkers for stratifying SLE risk. 

The advantage of assessing eGene expression is that it is affected by environmental factors. The polygenic risk score (PRS), which evaluates the effect of genome-wide polymorphisms based on GWAS data, has attracted great attention in risk assessment of disease [15]. The PRS is a useful method of risk assessment, but it has the drawback of not taking environmental effects into account. It was reported that assessment of eGene expression at the site of inflammation in inflammatory bowel disease was superior to a score determined based on whole-genome polymorphisms in terms of disease onset and severity [16]. This suggests that eGene expression levels, potentially reflecting environmental influences, contribute to disease stratification. This review will focus on such eGene expression and eGene-associated pathways in the risk stratification of lupus patients.

## 2. Associations between SLE Genetic Risk and Immune Cells

### 2.1. Functions of Disease Susceptibility Polymorphisms

GWAS is a method of genetic risk assessment developed in the 2000s, and via GWAS more than 3000 disease-susceptibility polymorphisms have recently been identified in immune-mediated diseases (https://www.ebi.ac.uk/gwas/) [17]. However, the effect of these disease-susceptibility polymorphisms on the immune system is unclear. It has been reported that only 10% of disease-risk polymorphisms are located in coding regions and alter amino acid sequences, while more than half are located in enhancers [18]. Since enhancers regulate the expression of their target genes, while most susceptibility polymorphisms in immune-mediated diseases do not alter the amino acid sequence but rather the gene expression level. In addition, enhancers are cell specific and function to induce cell-lineage-specific gene expression. This indicates that immune disease-susceptibility polymorphisms contribute to disease development by altering the expression of certain genes in specific immune cells, rather than in immune cells as a whole. This is very important when considering the pathogenesis of immune diseases, as it suggests that changes in gene expression in specific domains, rather than in the entire immune system (i.e., abnormalities in balance), lead to the development of immune diseases. In this context, information about the specific genes and immune cells affected by susceptibility polymorphisms is very important in understanding disease pathogenesis.

### 2.2. Functional Genome Database

Polymorphisms in enhancers or promoters that affect the expression of target genes, as described above, are referred to as eQTL. eQTL and their target genes in immune cells can provide major clues to understanding the function of disease-susceptibility polymorphisms, indicating a need for functional genome databases of eQTL in immune cells. In 2017, Ishigaki et al. reported a functional genome database consisting of transcriptome data and genetic polymorphisms from five immune cell subsets (CD4, CD8, B cells, NK cells, and monocytes) from 105 healthy individuals [19]. The following year, a group from the La Jolla Institute reported a functional genome database, called DICE, containing transcriptome and genetic polymorphism information from 13 subsets of CD4^+^ T cells derived from 91 healthy individuals [20]. Although DICE is a highly useful database, its limitation is that it does not include data from memory B cells nor neutrophils, nor from disease samples. In 2021, Ota et al. constructed a larger functional genome database, ImmuNexUT, which consists of transcriptome and genetic polymorphism information in 28 subsets of cells derived from 337 patients with immune-mediated diseases and 79 healthy individuals [21]. ImmuNexUT contains eQTL for 11,395 protein-coding genes and 3839 long noncoding RNAs, with an average of 7000 eQTL per subset. 

The eQTL identified in ImmuNexUT are the most distinctly functional among disease-susceptibility polymorphisms. Therefore, Ota et al. performed an LD score regression analysis by combining previously reported SLE GWAS data with ImmuNexUT eQTL data to evaluate the association between SLE genetic risk and immune cell subsets [21]. Naive B cells and unswitched memory B cells were significantly associated with SLE genetic risk, suggesting that B cells are the most important immune cells in terms of genetic risk. Of particular interest is the association of SLE with earlier B-cell differentiation stages (i.e., naive and unswitched memory B cells), rather than more advanced differentiation stages (i.e., plasmablasts and switched memory B cells). It is possible that B cells expressing self-reactive B-cell receptors at earlier differentiation stages may be involved in pathological conditions in SLE, such as consumption of complement.

### 2.3. Identification of eGenes in Each Immune Cell

Ota et al. combined the GWAS and ImmuNexUT data to identify eGenes in Japanese SLE patients [21]. Among 32 candidate susceptibility polymorphisms identified in GWAS of Japanese SLE, 20 exhibited eQTL effects in one or more subsets and were thus defined as eGenes (Table 1). Bentham et al. reported eGene data from European SLE-GWAS and immune cell gene expression [22], however, these do not overlap significantly with the results published by Ota et al. The reason for this may be the influence of ethnic differences, but it may also be due to differences in analytical methods. Three types of eQTL were observed in ImmuNexUT, those that exerted effects in: (1) a broad cell subset; (2) certain lineages such as T cells, B cells, and myeloid cells; and (3) specific cell subsets such as plasmablasts. This was thought to be due to the cell specificity of the enhancers associated with each eQTL. For example, ARHGAP31 is subject to eQTL effects only in plasmablasts. One polymorphism is in linkage disequilibrium with the SLE GWAS SNP rs36059542 in an open chromatin region specific to plasmablasts near the ARHGAP31 gene, and this may be the true susceptibility polymorphism.

## 3. Identification of Immune Pathways Associated with Organ Damage in SLE Using Functional Genome Data

Treatment of SLE involves glucocorticoids and immunosuppressive drugs, and the 5-year survival rate has improved to approximately 95% [23]. However, in many SLE cohorts, the median age of death is in the 60s, with a particularly high risk of death among those younger than 40 years [24]. Epidemiological studies of the causes of SLE-specific death have shown that the most common causes are cardiovascular disease, renal disease, infectious disease, and malignancy [4]. Organ damage in SLE is quantified using the SLICC/ACR damage index (SDI), and a high SDI in the musculoskeletal, cardiovascular, ocular, and neuropsychiatric systems was associated with death in cohort studies [25]. Since a high SDI is associated with glucocorticoids treatment, the principle of current SLE management guidelines is to reduce the dose of corticosteroids as far as possible [5]. Therefore, to stratify SLE patients according to prognosis, it would be useful to identify the immune pathways associated with SDI and to predict which patients are likely to have a high SDI.

Takeshima et al. evaluated the association between enhanced immune pathways and SDI in SLE, using cohort data linked to ImmuNexUT [11]. They found that the type I IFN pathway was upregulated in all immunocompetent cells, but there was no significant association between upregulation of this pathway and the SDI. Interestingly, the oxidative phosphorylation (OXPHOS) pathway was enhanced in B cells. Electron microscopy consistently revealed enlarged mitochondria in SLE memory B cells. It has been reported that stimulation of human B cells with a combination of type I IFN and a TLR9 ligand can promote plasma cell differentiation [26]. An in vitro experiment showed that stimulation of B cells with a TLR9 ligand, but not with IFNα, enhanced the OXPHOS pathway. This suggests that innate immune signaling in SLE is associated with enhanced OXPHOS pathway activity in B cells. Notably, the SDI was significantly increased in cases with enhanced OXPHOS pathway activity, suggesting that the OXPHOS pathway is associated with organ damage. Hydroxychloroquine (HCQ) inhibits TLR signaling and improves survival and renal damage in SLE, and guidelines recommend use of HCQ unless contraindicated [27]. Inhibition of TLR signaling by HCQ suppresses OXPHOS in B cells, which may be responsible for reduced organ damage and improved survival.

The association of SLE with genetic risk is a clue in assessing the link between the OXHOS pathway and SLE pathogenesis. SLC15A4, UBE2L3, LYST, and other genes associated with mitochondrial function have been identified as SLE eGenes in B cells. SLC15A4-knockdown cells demonstrated decreased mitochondrial membrane potential under starvation conditions [28]. As an E2 ubiquitin-conjugating enzyme, UBE2L3 regulates Parkin activation and mitophagy [29]. 

**Figure 1:**
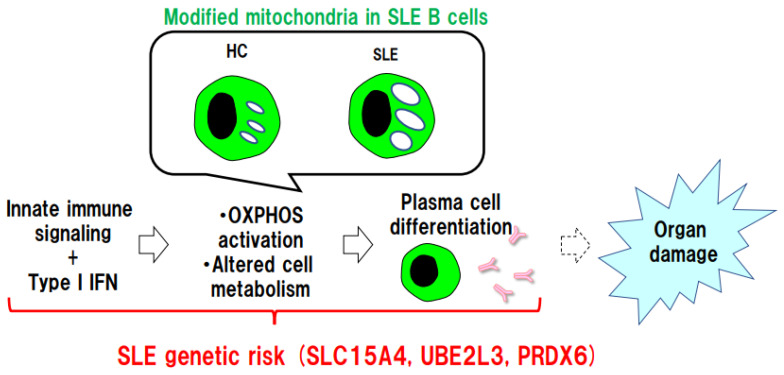
Activation of OXPHOS in B cells is associated with prognosis and genetic risk in SLE. Activation of OXPHOS in B cells, which may be induced by innate immune signals including TLR signaling, is associated with organ damage in lupus. Several SLE-susceptibility genes verified by eQTL analysis, SLC15A4, UBE2L3, and PRDX6, may play a role in this pathway.

Evaluation of gene co-expression networks has become a useful approach for organizing genes into groups or modules, based on expression correlations [30]. Highly correlated genes are likely to have similar functions and to transcriptionally regulate the same genes [31]. Disease-susceptibility genes whose expression regulates changes across a network of molecules involved in disease pathways are referred to as key driver genes. Takeshima et al. analyzed key driver genes in SLE and identified peroxiredoxin 6 (PRDX6) [11], which is an antioxidant enzyme that suppresses cellular ROS [32] and has been identified as an SLE-susceptibility gene [22]. In an eQTL analysis of Japanese subjects, PRDX6 was found to be an SLE-specific eGene in B cells. *Prdx6*-deficient B cells showed enlarged mitochondria and increased mitochondrial function. Moreover, *Prdx6*-deficient mice demonstrated exacerbated imiquimod-induced lupus. These findings suggest that OXPHOS is a genetic risk pathway for SLE (Figure 1).

## 4. Persistent OXPHOS Activation in Immune Cells in Inactive SLE Patients

SLE has a particularly high risk of death in the first few years after onset, and the increased risk persists throughout life. In a number of registry studies, 60% of SLE patients had low disease activity when taking low-dose steroids and immunosuppressive drugs. However, even under low disease activity, certain immunological pathways may remain elevated, leading to accumulation of organ damage [25]. Therefore, identification of the immune pathways responsible for high disease activity in SLE and those that remain activated under low disease activity may enable selection of effective therapies to improve long-term prognosis. Banchereau et al. analyzed gene expression in whole blood over time in pediatric SLE patients and found that the plasmablast signature was most strongly correlated with disease activity, and that the levels of neutrophil-specific transcripts increased as nephritis progressed [33]. Perez et al. profiled peripheral blood mononuclear cells using multiplexed single-cell RNA sequencing in 162 SLE patients and 99 controls [34]. Cell-type-specific expression predicted case–control status and identified patients prone to disease flares. However, the identified gene-expression modules demonstrated weak correlation with individual clinical features. Nakano et al. analyzed the immune pathways upregulated in 30 cases of SLE with high disease activity and 31 cases of inactive SLE (SLEDAI = 0) among 136 cases of SLE, using ImmuNexUT [35]. First, they compared SLE with high disease activity and inactive SLE and identified genes upregulated in the former; this gene signature was associated with the ribosomal pathway in all immune cells and with the cell-cycle pathway in T and B cells. Next, in a comparison of inactive SLE patients and healthy controls, they identified genes upregulated in inactive SLE, and reported that these genes were associated with the OXPHOS and mTOR pathways in B cells, as well as the complement pathway in all immune cells. These results indicate that the immune pathways that promote high disease activity in SLE differ from those that promote disease maintenance during the inactive phase. 

**Figure 2:**
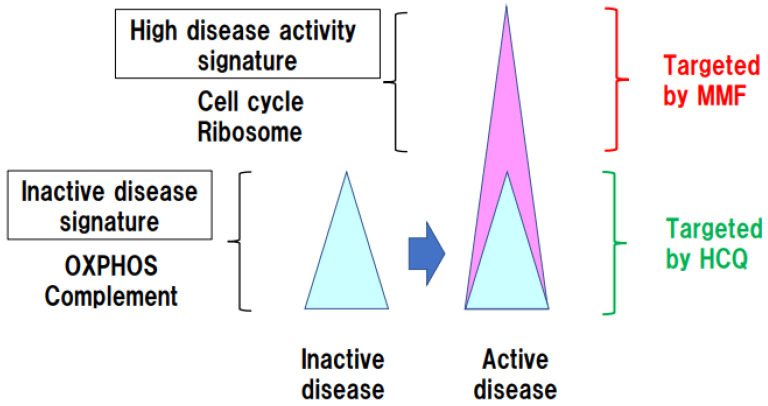
Two distinct signatures in SLE. Transcriptome analysis of SLE revealed that OXPHOS and complement pathways are the disease-state signatures upregulated in clinically stable SLE, and that the cell cycle and ribosomal pathways are the disease-activity signatures upregulated in active SLE. Interestingly, mycophenolate mofetil (MMF), an immunosuppressive drug effective in SLE, inhibited the cell-cycle pathway in the disease-activity signature. Hydroxychloroquine (HCQ) may suppress OXPHOS pathway in clinically stable SLE via inhibition of TLR signaling.

Immunosuppressive and molecular targeted drugs have been used in SLE, but the immune cell subsets and pathways that they target are unknown. ImmuNexUT analysis revealed that mycophenolate mofetil (MMF) suppresses genes associated with high disease activity, including cell-cycle-related genes in Th1 cells, central memory CD8^+^ T cells, and plasmablasts. Thus, it was hypothesized that SLE has an immunological structure in which the cell-cycle pathway associated with high disease activity is suppressed by MMF, and the residual OXPHOS pathway associated with non-active SLE is suppressed by HCQ (Figure 2).

## 5. Stratification of SLE Risk by SLC15A4-Regulated Histidine 

### 5.1. Function of the SLE eGene SLC15A4 

SLC15A4 is among the eGenes identified in ImmuNexUT, and displays eQTL effects in B cells. SLC15A4 is a lysosomal proton-binding amino acid transporter that translocates histidine and oligopeptides from within eukaryotic lysosomes to the cytosol. SLC15A4 is required for type I IFN production via TLR7 and TLR9 in plasmacytoid dendritic cells [36]. Loss of SLC15A4 in B cells disrupts endolysosomal pH regulation and activation of the mTOR pathway and prevents type I IFN production by IRF7 activation.

### 5.2. Histidine Depletion in SLE Plasma and SLC15A4 Function

Iwasaki et al. compared the metabolome of SLE patients with that of healthy controls and found that histidine levels were decreased in SLE patients and were associated with organ damage [37]. Furthermore, a decreased histidine level was not associated with type I IFN signaling, but was negatively associated with enhanced OXHPOS in plasmablasts. As histidine was found to be essential for plasmablast differentiation by type I IFN and innate immune signaling in experiments in vitro, it is possible that a decreased concentration of plasma histidine affects B-cell differentiation. As described above, the OXPHOS pathway remains active in inactive SLE [35], and the persistence of this immunological pathway in apparently disease-stable cases may be associated with poor long-term prognosis of SLE. Thus, metabolomic and transcriptomic analyses consistently demonstrate an association between OXPHOS and organ damage.

**Figure 3:**
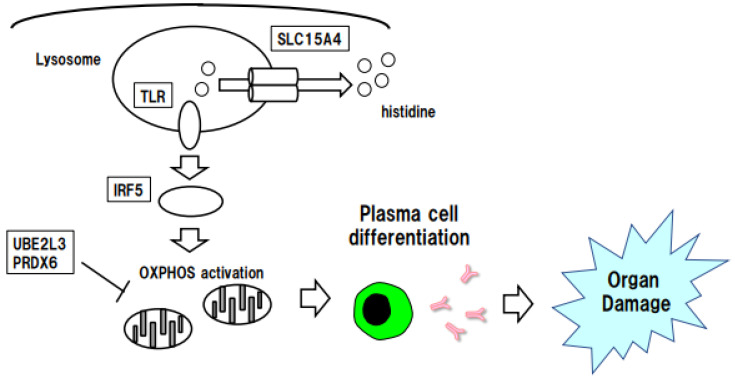
Possible mechanism of increased risk for SLE. Increased expression of SLC15A4 alters the intracellular level of histidine, enhances innate immune signaling by TLRs, enhances OXPHOS, and promotes B-cell differentiation, which is associated with organ damage.

ImmuNexUT eQTL analysis has shown that SLC15A4 expression is increased in individuals with SLE-risk genotypes. A possible mechanism for this is that increased expression of SLC15A4 alters intracellular levels of histidine, enhances innate immune signaling by TLRs, enhances OXPHOS, and promotes B-cell differentiation, which is associated with organ damage (Figure 3). Evaluation of OXPHOS pathway activity and SLE-risk genotypes for SLC15A4 may enable stratification of SLE cases prone to advanced organ damage.

## 6. Stratification of SLE Risk According to Immune Cell-Specific IRF5 Expression

### 6.1. Function of IRF5, an eGene for SLE

The SLE eGenes identified in ImmuNexUT include IRF5, a transcription factor that affects both innate and adaptive immunity. IRF5 is associated with the MyD88 adaptor protein downstream of TLR signaling via innate immune receptors and is activated by post-translational modifications such as ubiquitination and phosphorylation. Activated IRF5 is translocated from the cytoplasm to the nucleus, where it induces gene expression of inflammatory cytokines such as type I IFN [38,39]. Ban et al. reported that nuclear translocation of IRF5 was enhanced in monocytes from SLE patients [40]. They also showed that the mitochondrial dysfunction and disease status of an SLE mouse model were improved by deletion of IRF5. These results suggest that IRF5 is involved in mitochondrial dysfunction, as described above, and that IRF5 inhibition has a therapeutic effect on SLE.

The association between polymorphisms near the IRF5 gene and SLE has been reported in many GWAS. These polymorphisms occur at four main locations: two in the 5’ UTR, one in the 3’ UTR, and one approximately 5 kb downstream of the IRF5 gene. Of clinical importance is that the activity of serum type I IFN is increased in SLE that harbors both risk haplotypes in the IRF5 region and autoantibodies [41]. In ImmuNexUT, on the other hand, rs3757387 in the enhancer region of IRF5 exhibited the strongest eQTL effect in neutrophils, plasmacytoid dendritic cells, CD4^+^ T cells, and CD8^+^ T cells (https://www.immunexut.org/). Hou et al. reanalyzed the SLE GWAS results, focusing on enhancers near IRF5, and found that rs4728142 located within an enhancer in linkage disequilibrium with rs3757387 was associated most strongly with SLE [42]. They reported that the SLE-risk genotype of rs4728142 enhanced transcription factor ZBTB3, and deletion of the rs4728142 region in monocytes reduced the expression of IRF5, IL6, and IFNB. These findings suggest that polymorphisms within the enhancer region upstream of IRF5 are involved in SLE pathogenesis by affecting the expression of IRF5 and inflammatory cytokines in myeloid cells.

### 6.2. Linkage between IRF5 and OXPHOS

Functional and genetic links between IRF5 and TLRs have recently become apparent. TASL coded by CXorf21 on chromosome X interacts with SLC15A4 and is required for endolysosomal TLR response in immune cells [43]. TASL contains a conserved motif that mediates the recruitment and activation of IRF5. Notably, CXorf21 was reported to be a candidate gene underlying Xp21.2 SLE association, and the risk haplotype increased expression of TASL in lymphoblastoid cell lines (LCLs) [44]. Therefore, TLR-SLC15A4/TASL-IRF5 axis may support immune response and gender bias in SLE.

In addition to regulating the expression of proinflammatory cytokine responses to microbial and viral infection, evidence of IRF5 as a metabolic transcriptional regulator is emerging. In primary human monocyte-derived macrophages (MDMs), IRF5 was essential for pattern recognition receptor (PRR)-initiated upregulation of glycolysis [45]. Upon stimulation of the PRR nucleotide-binding oligomerization domain containing 2 (NOD2) in human macrophages, IRF5 binds RIP2, IRAK1, and TRAF6. Then, IRF5 contributes to optimal Akt2 activation, which increases expression of glycolytic pathway genes. IRF5 is a key component of airway macrophage metabolic responses following influenza infection and TLR3 activation [46]. IRF5 directly regulates metabolic genes such as hexokinase-2. Ban et al. reported that IRF5 regulated OXPHOS in a Lyn-deficient SLE mouse model [40]. IRF5 deficiency in Lyn-deficient mice specifically abrogated expression of OXPHOS-related genes. Upregulated mitochondrial membrane potential in the monocytes and pDCs of Lyn-deficient mice was significantly suppressed by IRF5 deficiency. In summary, IRF5 may be involved in the induction of OXPHOS genes, and contribute to mitochondrial dysregulation in SLE. 

### 6.3. Potential of IRF5 Gene Polymorphisms in Stratification of SLE Risk 

Type I IFN activity is increased in SLE sera, and the type I IFN gene is thought to originate from mitochondrial DNA. Mitochondria-containing neutrophil extracellular traps stimulate the cGAS/STING pathway in macrophages and TLR9 in plasmacytoid dendritic cells. Li et al. compared healthy individuals with the homozygous IRF5 SLE-risk haplotype and healthy individuals without this haplotype, and found that the former group had a higher rate of antinuclear and anti-SS-A antibody positivity, a higher proportion of plasma cells, and increased NETosis in neutrophils [47]. The expression and nuclear translocation of IRF5 in B cells did not differ between those with and those without the risk haplotype, suggesting that the IRF5-risk haplotype may act via its effect on myeloid cells.

Anifrolumab, an anti-IFNAR1 antibody targeting type I IFN, has been approved for SLE treatment. TULIP-2, a phase III study of anifrolumab, showed that it is particularly effective in cases with high IFN signatures [48]. In the general population harboring the IRF5 SLE-risk haplotype, serum type I IFN activity was reported to be high, while the risk of COVID-19 death was reduced [49]. Individuals with the IRF5 SLE-risk haplotype may have a better immune response to viral infection but may be at higher risk of developing SLE, an aspect of risk compensation. In the future, evaluation of IRF5 SLE-risk haplotypes and serum type I IFN activity may allow stratification of treatments including anifrolumab.

## 7. Conclusions

Although the development of PRS is a major advance, patient stratification by PRS alone may be difficult because therapeutic response and severity of common disease are significantly influenced by environmental factors. In addition to genomic information, highly accurate stratification of patients may be achieved by taking into account gene expression and immune cells that are influenced by the environment. Genes associated with genetic risk are particularly important, and eQTLs and eGene will provide fundamental information for precision medicine. Integrated analyses of GWAS and functional genome databases show potential for revealing the clinical significance of specific immune pathways in SLE. In particular, the B-cell OXPHOS pathway may be a stratification marker for organ damage in SLE. In the future, it is expected that the use of SLE-risk genotypes or haplotypes combined with immune-related gene expression will enable risk stratification of SLE.

## Figures and Tables

**Table 1:** List of eGenes for SLE-GWAS SNP.

	Bentham et al.	Ota et al.
	2015 [14]	2021 [13]
Chr	European	Japanese
1	FCGR2A	PTPRC
	FCGR2B	
	LYST	
2	IFIH1	LBH
3	ABHD6	ARHGAP31
	PXK	
4	BANK1	PAQR3
5		TCF7
		PTGER4
		GPX3
6	UHRF1BP1	
7	**IRF5**	**IRF5**
		NCF1
8	**BLK**	**BLK**
10	WDFY4	
11	IRF7	
12		SLC15A4
		APOLD1
13		ELF1
14		AHNAK2
15	CSK	RASGRP1
16	SOCS1	IRF8
	ITGAM	CDH1
		KAT8
19	TYK2	LRRC25
22	**UBE2L3**	**UBE2L3**

## Data Availability

No new data were created.
